# Rating of Perceived Exertion and Physiological Responses in Water-Based Exercise

**DOI:** 10.1515/hukin-2015-0112

**Published:** 2015-12-30

**Authors:** Stephanie Santana Pinto, Cristine Lima Alberton, Paula Zaffari, Eduardo Lusa Cadore, Ana Carolina Kanitz, Giane Veiga Liedtke, Marcus Peikriszwili Tartaruga, Luiz Fernando Martins Kruel

**Affiliations:** 1 Physical Education School, Federal University of Rio Grande do Sul (UFRGS), Porto Alegre, RS, Brazil; 2 Physical Education School, Federal University of Pelotas (UFPel), Pelotas, RS, Brazil; 3 School of Physical Education, Midwest State University of Parana, Guarapuava, Brazil

**Keywords:** minute ventilation, oxygen uptake, heart rate, electromyographic signal, aquatic exercise, floating equipment

## Abstract

The aim of the present study was to relate the overall rating of perceived exertion (RPE-overall) with cardiorespiratory and neuromuscular variables during stationary running with the elbow flexion/extension performed with water-floating equipment. The sample consisted of eleven women that performed the water-based exercise at submaximal cadences. The heart rate, oxygen uptake, ventilation, and electromyographic signal (EMG) from biceps brachii (%EMG BB), triceps brachii (%EMG TB), biceps femoris (%EMG BF) and rectus femoris (%EMG RF) muscles were measured during the exercise, and the overall RPE was measured immediately following its completion. The Pearson product-moment linear correlation was used to investigate associations between the variables analyzed in the present study. Significant relationships were observed between the RPE-overall and all the cardiorespiratory variables, with the r values ranging from 0.60 to 0.70 (p<0.05). In addition, the RPE-overall showed a significant (p<0.05) relationship with %EMG BB (r=0.55) and %EMG BF (r=0.50). These results suggest an association between the RPE-overall with all cardiorespiratory and two neuromuscular variables during the execution of a water-based aerobic exercise using water-floating equipment.

## Introduction

The intensity of water-based exercises may be manipulated by the use of different body shapes in the water (i.e., different exercises) and performing exercises using aquatic devices designed to take advantage of physical properties of water (i.e., drag and lift forces). There are two types of aquatic devices that are used in water-based exercises: water-floating and water-drag equipment (Colado and Triplett, 2009; De Souza et al., 2012). Previous studies have investigated cardiorespiratory and/or neuromuscular variables during water-based exercises (i.e., water-based aerobic and water-based resistance exercises) performed with and without aquatic devices (Colado et al., 2013; Pinto et al., 2006; Pinto et al., 2008; Pinto et al., 2011). The findings of most of these studies showed that the use of aquatic devices during performance of water-based exercises results in an increase in physiological responses when submaximal intensities (controlled cadences) are employed.

The intensity of water-based exercise programs has been defined by the heart rate (HR) (Takeshima et al., 2002; Tsourlou et al., 2006; Pinto et al., 2014) and the rating of perceived exertion (RPE) (Carvalho et al., 2009; Graef et al., 2010; Bento et al., 2012). The RPE is defined as the subjective intensity of effort, tension, discomfort and/or fatigue that is felt or experienced during aerobic and resistance exercises (Robertson and Noble, 1997). The use of the RPE could be prioritized as a measure for water-based exercise sessions due to its simple use, low cost and easy adaptation to aquatic activities (Alberton et al., 2011a). Moreover, its use is a comprehensive option for exercise intensity prescription for special populations who often make use of medications that interfere with the HR response and therefore, do not fit the criteria for exercise training using the HR as a parameter (Swank et al., 2003).

Studies of aquatic activities, such as shallow water walking, deep water running and water-based exercises, have found significant correlations between the RPE and cardiorespiratory parameters (Alberton et al., 2011a; Alberton et al., 2012; Pinto et al., 2013; Svedenhag and Seger, 1992). RPE response includes consideration of the tension on the neuromuscular system, which can be analyzed through the electromyographic signals (EMG) of muscle groups involved in the exercise. Although only the study of Alberton et al. (2011a) investigated the relationship between the overall rating of perceived exertion (RPE-overall) and the EMG signal of lower limb muscles during specific water-based aerobic exercise. The authors found no significant correlation between these variables, however, the local RPE (perceived exertion in lower limbs) was not analyzed.

To the best of our knowledge, no study has investigated the relationship between the RPE (overall and/or local) and cardiorespiratory and neuromuscular variables during performance of water-based aerobic exercises with water-floating equipment. It is known that aquatic devices are widely used in water-based classes in order to diversify and intensify them. In addition, the most commonly used devices in fitness centers are water-floating ones (Colado, 2004). As a result, it would be important to study the relationship between the RPE and cardiorespiratory and neuromuscular variables with the use of water-floating equipment, as this relationship could be changed by their use. Thus, the purpose of the present study was to relate the RPE-overall with cardiorespiratory and neuromuscular variables during stationary running with elbow flexion/extension performed with water-floating equipment. In addition, the correlation between the rating of perceived exertion in the upper (RPE-U) and lower limbs (RPE-L) with only neuromuscular variables during the exercise was assessed.

## Material and Methods

### Participants

Eleven physically active, healthy young women (age: 23.09 ± 2.07 years; body mass: 59.09 ± 4.84 kg; body height: 164.45 ± 5.54 cm; percent fat: 28.56 ± 2.95 %) volunteered for the present study. Subjects had practiced water-based exercises for at least 3 months, had no musculoskeletal, bone and joint, or heart or lung diseases, and were not taking any medication. Subjects were informed about the design of the study, as well as the possible risks and discomforts related to the measurements.

### Experimental design

In order to investigate the relationship between the RPE and physiological responses (i.e., cardiorespiratory and neuromuscular parameters) in water-based aerobic exercise performed with water-floating equipment, subjects attended to the laboratory on three different occasions. On the first day, the anthropometric measures were taken and a progressive maximal test was performed on a treadmill to obtain maximal oxygen uptake (VO_2max_) and heart rate (HR_max_) data. During the following session, the subjects were familiarized with the aquatic protocol that would be used. At the third session, the aquatic protocol was performed with the execution of stationary running with the elbow flexion/extension performed with water-floating equipment at two randomized cadences (80 and 100 bpm). During the performance of the water-based aerobic exercise, the HR, oxygen uptake (VO_2_), minute ventilation (V_E_), and EMG values from biceps brachii, triceps brachii, biceps femoris and rectus femoris muscles were measured. At the end of each cadence the overall and local body RPE were collected.

### Procedures

This study was approved by the Ethics Committee of the Federal University of Rio Grande do Sul and was performed in accordance with the ethical standards laid down in the Declaration of Helsinki (1964). An initial session was held with the collection of anthropometric measurements and performance of the maximal exertion test on a treadmill. Body mass and height were measured using an analogue medical scale and a stadiometer (Filizola, São Paulo, Brazil), respectively. Body composition was assessed using the skinfold technique (Lange, Cambridge, United Kingdom). The same experienced technician obtained all anthropometric measurements, on the right side of the subject’s body. A 7-site skinfold equation was used to estimate body density (Jackson et al., 1980), and body fat was subsequently calculated using the Siri equation (Siri, 1993). Afterwards, a maximal exercise test was carried out on a treadmill (Quinton, Seatle, USA) in order to evaluate VO_2max_ and the HR_max_. The protocol consisted of an initial velocity of 5km·h^−1^ with 1% inclination during 2 min. After this warm-up, the velocity was increased each minute by increments of 1km·h^−1^, and the inclination was maintained until the subjects reached their maximal effort. The assessment was considered valid when some of the following criteria were met at the end of the test (Howley et al., 1995): the estimated HR_max_ was reached (220-age), a plateau in VO_2_ with an increase in the treadmill velocity and a respiratory exchange ratio greater than 1.15 was reached.

After the initial session, the subjects were familiarized with stationary running with the elbow flexion/extension, intensities and equipment that would later be used during data collection. The full range of motion during the execution of the exercise was described to the participants. In addition, all the instructions about the RPE scale were also explained. The Borg 6–20 RPE scale is an instrument for determining rates of perceived exertion developed to enable reliable and valid estimates of perceived exertion (Borg, 1990). To familiarize the subjects with the use of the scale, the water-based exercise was performed in water at all effort levels progressively to familiarize them with the minimal effort and graduation until the maximal effort by their self-pace. Subjects were instructed to try to understand the degree of tension and fatigue in their muscles, shortness of breath, or chest pain.

During the last session the subjects performed the aquatic protocol with the data collection of physiological variables. The following protocol was applied: performance of the maximal isometric voluntary contraction (MIVC) on land and execution of the water-based exercise protocol. The water-based exercise was performed with water-floating equipment at two submaximal cadences (80 and 100 bpm) that were reproduced by a digital metronome (MA-30, KORG; Tokyo, Japan) and were performed randomly with a 5 min interval between them. Each segmental action (hip flexion or extension and elbow flexion or extension) was performed in one beat. The subjects performed the exercise for 4 min at each cadence.

In order to collect the EMG data during the aquatic protocol the preparation of subjects skin was performed. Hair was removed from the surface of the muscles, which were then cleaned by rubbing with cotton wool dipped in alcohol for the subsequent placement of the electrodes on the short head of biceps brachii (BB), lateral head of triceps brachii (TB), rectus femoris (RF) and long head of biceps femoris (BF). The innervation zone of each muscle of the right arm and leg was determined with the aid of an electrostimulator (EGF 4030, CARCI, São Paulo, Brazil). Bipolar electrodes (20 mm inter-electrode distance) were placed 2 cm distal from the innervation zone. The inter-electrode resistance level regarded as suitable was below 3,000 ohms. The reference electrode was positioned on the clavicle. An insulation procedure was performed in order to avoid interference due to the contact of electrodes with water, which could produce noise in the EMG signal (Rainoldi et al., 2004). Silicone glue was placed at the exit point of the cables (dried for approximately 1.5 h) to prevent water from entering. The cables and preamplifiers were fixed with adhesive tape. Previous studies found that these insulation procedures did not interfere with the EMG signal (Carvalho et al., 2010). The EMG data were collected using a 14-bit electromyograph (Miotec, Biomedical Equipment, Porto Alegre, Brazil), with a 4-channel system at 2,000 Hz per channel. After the preparation of subjects skin, a 5 s MIVC was performed in each involved joint in order to estimate the maximal EMG amplitude for each muscle (i.e., BB, TB, RF, and BF). The MIVC values were used for further normalization of the EMG signal (Knutson et al., 1994). Pinto et al. (2010) had previously described the MIVC procedures.

After the CIVM performance, the subjects started the water-based aerobic exercise. The initial position was in upright with arms hanging down at the sides of the body. The first phase of the movement consisted of right hip and knee flexion at 90° and right and left elbows flexion at 90° with supine radio-ulnar, simultaneously. The second phase consisted of full right hip and knee extension and right and left elbows extension with supine radio-ulnar. This movement was repeated alternately with the right and left lower limbs, and visual feedback was given to the subjects in order to maintain the range of motion during the data collection. The equipment was positioned 3 cm above the lateral malleolus on the lower limbs, and in the upper limbs they were attached in the middle of the hand (hand-grip). The water-floating device (hydro floating arm and leg trainer, Aktiv, Brazil) was used like a buoyancy device with vertical displacements of both upper and lower limbs. Subjects were immersed between the xiphoid process and shoulders. The water temperature was maintained at 30.80 ± 0.42 °C. The following instructions were given to the subjects for experimental protocol completion: fast for a period of 3 to 4 h before the test session, do not ingest stimulants, hydrate at will, and avoid performing intense exercises during the last 24 h. The sessions were always held in the afternoon, between 4:00 and 7:00 PM.

In order to evaluate VO_2_ and V_E_, a mixing-box-type portable gas analyzer (VO2000, Inbramed, Porto Alegre, Brazil) was used. The gas analyzer was calibrated prior to each collection session according to manufacturer’s specifications. The HR was measured using a Polar monitor (FS1, Shangai, China). The sampling rate of the collected VO_2_ and the HR was 10 s. During the exercise the mean value (3^rd^ to the 4^th^ min) for the HR and VO_2_ was obtained for each intensity. Also, HR and VO_2_ values were expressed as percentages of the HR_max_ (%HR_max_) and VO_2max_ values (%VO_2max_). The RPE-overall, the RPE-U and RPE-L were measured at the end of the exercise in each cadence using the Borg 6–20 RPE scale (Borg, 1990). The scale (banner, 60 × 90 cm) was fixed out of the pool in front of the subjects.

In order to determine the angular position of the elbow and the hip over the range of motion for the alignment of the EMG signal during the exercise, reflexive markers were placed on the middle point between acromion and lateral epicondyle of the humerus, lateral epicondyle of the humerus, styloid process of the radius, greater trochanter of the femur and lateral epicondyle of the femur for filming in order. A video camera, which was positioned in the sagittal plane on the right side of the subjects, at a distance of 3 m, was used to obtain the angular position for the elbow and the hip. The film was shot through an underwater window in the side of the pool using a video camera (JVC GR-DVL9800, Yokohama, Japan). The kinematical data procedures were previously described by Alberton et al. (2011b). In order to synchronize the EMG data with the angular position of the elbow and the hip, a light signal was triggered with the onset of EMG data collection.

Following acquisition of the signal, the data were exported to the SAD32 software (Mechanical Measurements Laboratory, Federal University of Rio Grande do Sul, Porto Alegre, Brazil) where they were filtered using a fifth-order band-pass Butterworth filter, with cut-off frequencies between 20–500 Hz. The signal curves corresponding to the MIVC (5 s) were sliced between 2 and 4 s to obtain the root mean square (RMS) value. Based on an angular position, the starting and finishing time points of each repetition were used to make slices of each total repetition of the exercise (hip and elbow) of each subject for the first 5 repetitions of the acquired signal. The RMS value corresponding to each 1 of the 5 repetitions was obtained and a mean of the 5 repetitions was made. These values were normalized and expressed as a percentage of the maximal EMG obtained during the MIVC (%EMG).

### Statistical analysis

Normality of the data distribution was tested using the Shapiro-Wilk’s test. Pearson product-moment linear correlation was used to check the level of the relationship between the cardiorespiratory and neuromuscular variables with the RPE-overall and to verify the relationship between only the neuromuscular variables with the local RPE (i.e., RPE-U and RPE-L). Multiple linear regression was carried out using the Forward method to verify the contribution of the variables to explain the RPE- overall. An alpha level of 0.05 was adopted for all statistical tests, which were performed using the SPSS software (version 19.0).

## Results

Significant relationships were observed between the RPE-overall and all the cardiorespiratory variables, with the r values ranging from 0.60 to 0.70 and p<0.05. In addition, the RPE-overall showed a significant (p<0.05) relationship with the %EMG BB (r=0.55) and %EMG BF (r=0.50). The %EMG TB (r=0.42, p=0.072) and %EMG RF (r=0.37, p=0.177) showed no significant correlation with the RPE-overall.

**Figure 1:**
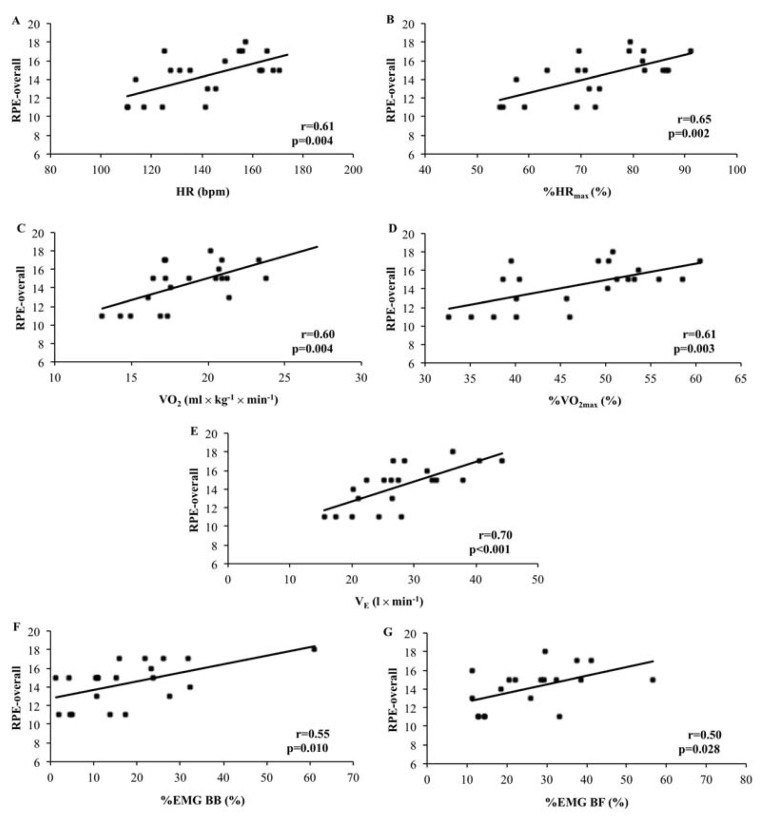
The relationship between rating of perceived exertion overall (RPE-overall) and the heart rate (HR) (A), RPE-overall and percentage of the maximal HR (%HR_max_) (B), RPE-overall and oxygen uptake (VO_2_) (C), RPE-overall and percentage of the maximal VO_2_ (%VO_2max_) (D), RPE-overall and ventilation (V_E_) (E), RPE-overall and percentage of maximal muscle activation of biceps brachii (%EMG BB) (F) and RPE-overall and percentage of maximal muscle activation of triceps brachii (%EMG TB) (G).

Significant relationships between the RPE-overall with cardiorespiratory and neuromuscular variables are presented in Figure 1. The EMG values from the BB, RF, RF and BF muscles showed no correlation with the local RPE (RPE-U and RPE-L) (Table 1).

Multiple linear regression was performed to identify which variables could best explain the RPE-overall. Based on the analysis carried out using the Stepwise method, it was concluded that the regression model was significant (p<0.001) and presented an r^2^=0.72, whereas the variables that best explained the RPE-overall were VE and %EMG BB. These results are showed in Table 2.

## Discussion

The primary findings of this study were the positive and significant relationships between the RPE-overall and cardiorespiratory parameters. In addition, the multiple linear regression showed a significant model and presented an r^2^=0.72, while the variables that best explained the RPE-overall were V_E_ and %EMG BB. No significant relationships were observed between the local RPE and neuromuscular variables.

The positive and significant relationships found in the present study between the RPE-overall and cardiorespiratory variables corroborate with studies which also investigated aquatic exercises. Alberton et al. (2012) investigated three pooled water-based aerobic exercises (stationary running, frontal kick, jumping jacks) and showed significant relationships between the RPE-overall and VO_2_ (r=0.65) and the RPE-overall and %VO_2max_ (r=0.68). In the study developed by Raffaelli et al. (2012), significant positive correlation (r=0.44) was found between the RPE-overall and VO_2_ in five pooled water-based aerobic exercises (stationary running, frontal kick, cross-country skiing, jumping jacks and lateral kick). In the present study significant relationships between the RPE-overall and VO_2_ (r=0.60) and the RPE-overall and %VO_2max_ (r=0.61) were observed during a water-based aerobic exercise executed with water-floating equipment in both upper and lower limbs. Shono et al. (2000) found a significant correlation between the RPE-overall and the HR during underwater treadmill walking (r=0.99). The results of our study showed significant correlations between the HR (r=0.61) and the %HR_max_ (r=0.65) in relation to the RPE-overall. The study of Alberton et al. (2011a) was the only one which analyzed several cardiorespiratory variables, and reported significant correlations between the RPE-overall and different physiological parameters during water-based stationary running exercise (HR: r=0.65, %HR_max_: r=0.65, VO_2_: r=0.60, %VO_2max_: r=0.71, V_E_: r=0.77). It is important to highlight that the present study also analyzed the relationships between the RPE-overall and several cardiorespiratory parameters during the stationary running with the elbow flexion/extension performed with water-floating equipment.

Regarding the use of aquatic devices, only one study was found in the reviewed literature that analyzed the utilization of water-based drag equipment (Pinto et al., 2013). In this study, significant correlations were observed between the RPE-overall and cardiorespiratory parameters during the execution of a water-based cross-country skiing exercise with water-drag equipment on the lower limbs (HR: rho=0.71, %HR_max_: rho=0.70, VO_2_: rho=0.60, %VO_2max_: rho=0.85, V_E_: rho=0.88). However, when the water-based drag equipment was used in both upper and lower limbs a significant correlation was only found for the RPE-overall and V_E_ (rho=0.71). In the present study, in which stationary running with elbow flexion/extension was performed with water-floating equipment in both upper and lower limbs, significant and positive correlations were observed between the RPE-overall and all evaluated cardiorespiratory variables (r=0.60–0.70).

Significant correlations for the relationships between the RPE-overall and neuromuscular variables were seen between the RPE-overall and %EMG BB (r=0.55) and the RPE-overall and %EMG BF (r=0.50). This result was partially different from the observations in the study of Alberton et al. (2011a) which evaluated the relationship between the RPE-overall with the %EMG from the rectus femoris, vastus lateralis, short head of biceps femoris and semitendinosus muscles during stationary running performed in the aquatic environment at three submaximal cadences. The authors observed no significant relationship between any of them. However, in this study, a different methodological approach was employed as the upper limbs were used only to maintain corporal balance and no aquatic device was used during the performance of the exercise.

In the present study, the absence of a correlation between the RPE-overall and %EMG TB could most likely be explained by a disproportional increase in the local stimulus provided by the use of water-floating equipment against lift force during the elbow extension. In contrast, a significant correlation between the RPE-overall and %EMG BB could most likely be explained by the neuromuscular activity of the elbow flexor being proportional to the RPE-overall, because lift force assists the movement. Thus, the relative intensity elicited by the dumbbells during the extension is greater than during the flexion of the elbow.

For the lower limbs, it was verified that there was no relationship between the RPE-overall and %EMG RF. This lack of significant correlation may be explained by the lower neuromuscular activation compared to the overall exercise intensity. Although the intensity of the exercise increases with the increments in cadences, as observed in the mean RPE-overall ranging from 13 to 16, it was not sufficient to increase the neuromuscular activation, observed through the mean %EMG RF values that varied from 10.5 to 16.6%. In contrast, the significant correlation found between the RPE-overall and %EMG BF could be explained by a proportional increase in neuromuscular activation (%EMG BF ranging from 19.7 to 33.4%) and the RPE-overall. It is important to note that the lower limb muscles evaluated are biarticular and performed actions both towards and against the lift force, in contrast to the muscles of the upper limbs (i.e., monoarticular muscles). Another aspect that must be emphasized is that the overload applied on the upper limbs seems to be greater compared to the lower limbs. This fact may be speculated because the size of water-based floating equipment was proportionally higher on the upper limbs when compared to the equipment used on the lower limbs, considering the difference in the muscles groups size (i.e., greater in the lower limbs).

Regarding the regression, the r^2^ explained 72% of the RPE-overall and 28% could be explained by other variables that were not analyzed in the present study. From the analysis, the variables that best explained the model were VE and %EMG BB. Thus, the results are in line with the definition of perceived exertion, which has an important pulmonary component, as demonstrated by the fact that the V_E_ had the best relationship with the RPE-overall, and a musculoskeletal system component, represented by the %EMG BB. Alberton et al. (2011a) also reported through linear multiple regression that the V_E_ was the main variable that explained the RPE-overall associated with the %VO_2max_ (r^2^=0.79).

For the local RPE, no significant correlations were found between the RPE-U and the muscles of the arm (BB and TB) and between the RPE-L and the muscles of the thigh (RF and BF). These results may be explained by the methodology used in the collection of the neuromuscular activation data (i.e., surface electromyography) as there are more active fibers than those that an electrode is able to capture (De Luca, 1997). In addition, the muscle activation of other heads from BB and TB and/or other synergistic muscles was not measured. Moreover, it is important to note that the water-based exercise was performed over 4 min, with a predominant aerobic component, in contrast to the water-based resistance exercise. Thus, the above-mentioned factors most likely explain the disproportional relationship between the local RPE and muscle activation.

The present results showed an association between the RPE-overall with all cardiorespiratory and two neuromuscular variables during the execution of a water-based aerobic exercise using water-floating equipment. No relationships were verified between the local RPE and neuromuscular variables. However, caution is necessary in the interpretation of these results, as the present study investigated a low sample size that was comprised of nine young women only. In addition, only one type of water-based exercise was analyzed (i.e., stationary running). The fact that different synergistic muscles were not evaluated during the performance of stationary running with elbow flexion/extension and the fact that this exercise was not performed at higher cadences of movement could be limitations of the study. Thus, the results of the present study suggest that the overall rating of perceived exertion could be used as an indicator of aerobic intensity during stationary running with elbow flexion/extension performed with water-based floating equipment for young women in water-based sessions. The advantages associated with the use of the RPE are its ease of use, low-cost and easy adaptation to water-based group sessions. However, it is important to note that suitable instructions and introductory sessions in the use of the scale are fundamental to ensure effective results.

## Figures and Tables

**Table 1:** The r and p values for the relationship between the rating of perceived exertion for the upper limbs (RPE-U) and the percentage of maximal muscle activation from the biceps brachii (%EMG BB), the RPE-U and the percentage of maximal muscle activation from the triceps brachii (%EMG TB), the rating of perceived exertion for the lower limbs (RPE-L) and the percentage of maximal muscle activation from the rectus femoris (%EMG RF) and the RPE-L and the percentage of maximal muscle activation from the biceps femoris (%EMG BF)

	r	*p*
RPE-U - %EMG BB	0.32	0.154
RPE-U - %EMG TB	0.34	0.159
RPE-L - %EMG RF	0.08	0.785
RPE-L - %EMG BF	0.41	0.084

**Table 2:** Coefficient beta (β) and p values for the variables ventilation (V_E_) and the percentage of maximal muscle activation of biceps brachii (%EMG BB) that entered in the model to explain the rating of perceived exertion overall.

	β	*p*
Constant	7.775	<0.001
V_E_	0.189	<0.001
%EMG BB	0.061	0.013
